# If Integration Is the Answer, What Was the Question? What next for English Health and Social Care Partnerships?

**DOI:** 10.5334/ijic.2535

**Published:** 2016-10-28

**Authors:** Jon Glasby

**Affiliations:** School of Social Policy, Muirhead Tower, Room 829, University of Birmingham, Edgbaston, Birmingham, B15 2TT, GB

‘Integrated care’ has been a key policy priority in English health and social care in recent years – although the extent to which this commitment is real rather than rhetorical remains open to debate. While everyone working in health and social care knows that single agency responses are insufficient when trying to support people with complex needs, there is a danger that ‘integrated care’ becomes a buzzword, apparently capable of resolving a range of different longstanding policy problems at once. This is particularly the case in a very challenging financial climate, when some policy makers appear to assume that this way of working will be able to dramatically improve outcomes whilst also significantly reducing costs. Such is the faith in integrated care, indeed, that it has even spawned its own policy joke: if you rang up some policy makers in the middle of the night to say that your house was on fire (so the joke goes), they would say: “that’s a shame – what you need is more integrated care!”

Despite all this, some of the initiatives being developed to promote more integrated care seem remarkably similar to those pursued by the New Labour government of 1997–2010 – whether this is pooled health and social care funds, joint health and social care governance arrangements, integrated teams, or local pilots to produce rapid policy learning. However, one of the key lessons throughout this period is that local services seeking to integrate care can find it very difficult to do so in a system not designed with integration in mind. While different areas of the country have made progress at different times and in different ways, longstanding barriers to joint working, a rapidly changing policy context, significant central control and the emergence of other single agency priorities over time have all made it difficult to join services up in practice.

Against this background, both research and recent practical experience suggest one thing that probably will not work, and four things that might. Often, debates about integrating care can focus on integrating separate organisations, and the English NHS in particular is reorganised on a regular basis. While such structural ‘solutions’ look dramatic and bold, the evidence around mergers and acquisitions (in both public and private sectors) is that they rarely achieve stated objectives, often fail to save money and tend to reduce morale, productivity and positive service developments (often for some 18 months to two years after the initial change – if it is managed well). Moreover, the evidence from the NHS is that hospital mergers tend not to take place for the reasons stated in the consultation document – but in response to local/national politics, to save money (even though they tend not to) and to get rid of management teams that are deemed to be failing. None of this is to say that changing structures cannot be part of a broader solution; it is just that it often isn’t – and it certainly should not be the first place to start.

More fruitful might be some or all of the following four approaches:

1. Being very clear about the ***outcomes*** that partners are trying to deliver. This sounds deceptively simple, but public services in particular can often start to talk about outcomes, but accidentally drift back into debates about processes and structures. Although partners often argue about what they should do at local level, these issues ought to more straightforward if we had a genuinely shared understanding of where we are now and of where we are trying to get to. Without a shared sense of what success would look like, we can have no basis for deciding how best to design local joint working arrangements and no way of knowing if our chosen approach is working. The next time a new national policy initiative comes along, moreover, we will have no way of receiving it, making sense of it for local people and implementing it in a way that discharges our responsibilities, but which also furthers our goals. It is this that lies behind the sub-title of this paper: if integration is the answer, what was the question?
2. Successful joint working depends on working with different ***professional values and cultures***. Neglecting this can make the practitioners involved fearful that their professional status and identity may be undermined, and they tend to pull back from each other – more jealously guarding what makes one person a social worker and the other person a nurse (as but one example). It then takes significant time before workers feel sufficiently safe and comfortable to take several steps forward and to meet the other person half way, sharing what they do and learning from others. Although much less dramatic than creating new structures, focusing on organisational and professional culture is crucial – and we neglect this at our peril.
3. Adult social care in England is currently embedding a **‘*personalisation*’** agenda, which includes giving people using services greater control over the care they receive via direct payments and personal budgets. This is also being developed in the NHS, albeit at a smaller scale and a slower pace and with greater cultural barriers to overcome. In many areas of the country, partnership working and personalisation are seen as separate ways of working that are being promoted in potential isolation from each other. However, a different approach would be to see these two agendas as two sides of the same coin, enabling people with complex needs to join up their own care and support across traditional agency boundaries in a way that makes sense for them. This is a much more bottom-up notion of integration than previous, top-down attempts to integrate management teams or budgets – and might just prove more promising.
4. Last but not least, we have previously argued that local government (which is responsible for social care) and the NHS (responsible for health care) need each other now more than ever. Local government has been tasked with promoting social and economic well-being and with being a strategic place-shaper (the one organisation locally responsible for identifying and nurturing the unique nature of an area). To do this, it needs a strong relationship with the NHS, since local health services are so important to local people. At the same time, the NHS has to take some very difficult decisions (for example, about issues such as acute care reconfiguration) – and needs to learn from the best of local government. It is often said that the NHS is good at making the ‘right’ decision (i.e. in a logical, rational way), but that local government is much better at making decisions in the ‘right’ way (that is, paying attention to the politics of change and engaging local people in such a way as to maximise the chances of successful implementation). In one sense, this is as much about local identity and legitimacy as it is about the actual decision that has been taken – and local government tends to be part and parcel of people’s sense of local identity in a way that is difficult for health services (whose boundaries frequently change). As a result of all this, the time may be right for ***a more fundamental reconsideration of the relationship between health and social care***, rather than additional pilot projects and initiatives (which can only ever take us so far). One example of this might be exploring scope for more Scandinavian-style local government-led health care, changing the accountabilities of local health care so that it is much more responsive to the needs of local people and less influenced by central control. Another approach might be to make adult social care free at the point of delivery, funded from general taxation – so that both health and social care are financed in the same way with fewer financial barriers to joint working. While different people would agree or disagree with such proposals, the point is that something more fundamental might be required to genuinely achieve integrated care.

Above all, integration should be a means to an end of better services and better outcomes for people’s lives. If it somehow becomes an end in itself then we have lost sight of something important, and integration has become part of the problem rather than part of the solution.

## Author Information

Jon Glasby is Professor of Health and Social Care and Head of the School of Social Policy at the University of Birmingham. This commentary is based on a public lecture given as part of Edge Hill University’s ‘I4P’ lecture series in April 2016, in conjunction with the International Federation of Integrated Care. It draws on Glasby and Dickinson’s (2014) *Partnership working in health and social care: what is integrated care and how can we deliver it* (2^nd^ ed.). Bristol, The Policy Press.

